# Finasteride treatment and male breast cancer: a register‐based cohort study in four Nordic countries

**DOI:** 10.1002/cam4.1273

**Published:** 2017-12-13

**Authors:** Mathias Meijer, Lau Caspar Thygesen, Anders Green, Martha Emneus, Klaus Brasso, Peter Iversen, Eero Pukkala, Kristian Bolin, Knut Stavem, Annette K. Ersbøll

**Affiliations:** ^1^ National Institute of Public Health University of Southern Denmark Copenhagen Denmark; ^2^ Department of Nursing Metropolitan University College Copenhagen Denmark; ^3^ Institute of Applied Economics and Health Research Copenhagen Denmark; ^4^ Odense Patient Data Explorative Network Odense University Hospital and University of Southern Denmark Odense Denmark; ^5^ Copenhagen Prostate Cancer Center and Department of Urology Rigshospitalet Copenhagen Denmark; ^6^ Finnish Cancer Registry Institute for Statistical and Epidemiological Cancer Research Helsinki Finland; ^7^ School of Health Sciences University of Tampere Tampere Finland; ^8^ Department of Economics University of Gothenburg Gothenburg Sweden; ^9^ Centre for Health Economics at the University of Gothenburg Gothenburg Sweden; ^10^ Health Services Research Unit Akershus University Hospital Oslo Norway; ^11^ Department of Pulmonary Medicine Medical Division Akershus University Hospital Oslo Norway; ^12^ Institute of Clinical Medicine Faculty of Medicine University of Oslo Oslo Norway

**Keywords:** Breast neoplasms male, finasteride, Nordic countries, pharmacoepidemiology, prostatic neoplasms, registers

## Abstract

A potential link has been suggested between dispensed finasteride and increased risk of male breast cancer (MBC). Due to the rare occurrence of MBC, it remains to be established if such a relationship exists. The purpose of this study was to combine nationwide registers in four countries to assess the potential association between dispensed finasteride and MBC. A cohort of all males with dispensed finasteride in Denmark, Finland, Norway, and Sweden (1,365,088 person years) was followed up for up to 15 years for breast cancer, and compared to a cohort of males unexposed to finasteride. Individual‐level register data included country, dates of dispensed finasteride, MBC diagnosis, and death. Incidence rate ratios (IRRs) were estimated using a generalized linear model with a Poisson distribution. An increased risk of MBC was found among finasteride users (IRR = 1.44, 95% confidence interval [95% CI] = 1.11–1.88) compared to nonusers. The IRR increased to 1.60 (95% CI = 1.20–2.13) when users in Norway and Sweden with short follow‐up time were excluded. The highest IRR was seen among men with medium duration of dispensed finasteride, medium accumulated consumption of finasteride, and among men with first dispensed finasteride prescription 1–3 years prior to diagnosis. The analyses suggested possible ascertainment bias and did not support a clear relationship between dispensed finasteride and MBC. In conclusion, a significant association between dispensed finasteride and MBC was identified. However, due to limited data for adjustment of potential confounding and surveillance bias in the present study, further research is needed to confirm these results.

## Introduction

Finasteride is used primarily to relief symptoms from benign prostatic hyperplasia (5 mg pills) and to treat androgenetic alopecia (1 mg pills). Finasteride inhibits the type 2 5*α*‐reductase enzyme, thereby inhibiting the conversion of testosterone to dihydrotestosterone, leading to shrinkage of androgen‐dependent prostate tissue 1. Use of finasteride increases estradiol levels, which may cause gynecomastia 2. Increased estradiol levels caused by finasteride have raised concerns about a possible link between finasteride use and male breast cancer (MBC) 3, 4, 5, 6, 7, 8. In a cross‐national study in Denmark, Finland, Norway, and Sweden, dispensed finasteride was estimated at 10.8 per 1000 men in 2009 9.

A review found 50 spontaneously reported MBC cases worldwide in patients treated with 5 mg finasteride and three reports in patients treated with 1 mg finasteride 6. The median time to onset of MBC after starting 5 mg finasteride treatment was 36 months (range: 5 weeks to 11 years). The review identified eight cases that were observed in three placebo‐controlled clinical trials 4, 5, 8, where individuals were exposed to 5 mg finasteride and followed up for up to 5 years, altogether for 89,496 person years (PY). The crude incidence rate of MBC in persons treated with finasteride was 7.8 per 100,000 PY (95% confidence interval [95% CI] = 3.7–16.4), which was higher although not statistically different from the MBC rate 3.8 per 100,000 PY (95% CI = 1.2–11.9) in the unexposed group (*P* = 0.33). On this basis, the review concluded that it could not be excluded that finasteride could be associated with an increased risk of MBC 6. Two recent studies found no significant association between finasteride use and MBC 10, 11.

MBC is a rare disease with a reported age‐adjusted incidence rate of 0.4–1.0 per 100,000 PY in Europe 12, 13, 14. Investigating the possible link between finasteride use and MBC is challenging due to the combination of a limited number of MBC cases 15, 16, 17, 18, 19, 20, 21, 22, potential surveillance bias due to the association between finasteride use and breast enlargement and tenderness, and confounding from obesity, alcohol intake, liver disease, and family history 23. The objective of this study was to investigate if an association exists between dispensed finasteride and MBC incidence in Denmark, Finland, Norway, and Sweden using register data containing all dates and amounts of finasteride redeemed from pharmacies.

## Methods

### Study design

A register‐based cohort study with prospective data collection was used including all males in Denmark, Finland, Norway, and Sweden. Dispensed finasteride was the exposure variable and MBC diagnosis the study outcome. In this study, the term dispensed finasteride means that finasteride has been purchased and handed out to the patient. The incidence rate of MBC was compared between an exposed cohort of all men in the four Nordic countries having redeemed finasteride and a cohort of all men in the Nordic countries unexposed to finasteride.

### Data sources and study period

Data were extracted from the national prescription, cancer, and cause of death registers in the four countries. All redeemed prescriptions of finasteride were extracted for 1995–2009 in Denmark, 1997–2010 in Finland, 2004–2009 in Norway, and 2005–2009 in Sweden where finasteride is a prescription‐only medicine. The Anatomic Therapeutic Classification (ATC) code is D11AX10 for 1 mg finasteride and G04CB01 for 5 mg finasteride (in Finland in 1994–1996, 5 mg finasteride was registered as ATC G04CB04 and was not included in the study). During the study period, 1 mg finasteride tablets were sold only in Denmark and Sweden. All inhabitants in the Nordic countries have a unique personal identification code (PIC), which facilitate linkage of individual‐level register data. The PIC is given at birth or immigration, and is used as the identification key in the national registers. Individual information on dates of MBC diagnoses was extracted from national cancer registers. Only the first male breast cancer tumor was included. Individual information on date of death was extracted from the national cause of death registers.

Information for the general populations on the number of MBC cases and the size of the population was obtained from the NORDCAN database for each country and calendar year (1995, 1996, …, 2010) stratified by 5‐year age groups (0–4, 5–9, 10–14, …, 80–84, ≥85 years) 21. NORDCAN is a Nordic database and program on the incidence, prevalence, mortality, and survival statistics from 50 major cancers in the Nordic countries. Data in each of the Nordic countries are available for more than five decades. NORDCAN provides access to summary data with graphic and tabulation facilities.

### Measures

The exposed cohort consisted of all men with a dispensed finasteride prescription in the study period. Individual‐level data were obtained for the exposed cohort from the prescription registers including information on PIC, date of dispensed finasteride prescription, age of the recipient number of packages purchased, pills per package, and dose per pill. These data were linked with individual information on dates of MBC diagnoses from national cancer registers and with individual information on date of death from the national cause of death registers. For the exposed cohort, follow‐up started on the date of the first dispensed finasteride. End of follow‐up was the date of first MBC diagnose, date of death, date of emigration, or end of the study (31 December 2009 in Denmark, Norway, and Sweden, and 31 December 2010 in Finland), whichever came first. The number of PY and number of men with MBC in the exposed cohort were calculated in 5‐year age groups (i.e., 0–34, 35–39, 40–44, …, 80–84, ≥85 years), country, and calendar year.

The unexposed cohort was established as the difference between the general population and the exposed cohort. This was done by calculating PY in the unexposed cohort as the difference between the size of the general population and the PY in the exposed cohort by each 5‐year age group (i.e., 0–34, 35–39, 30–44, …, 80–84, ≥85 years), country, and calendar year. Hereby, men with a dispensed finasteride prescription contributed with nonexposed risk time before date of first dispensed finasteride prescription and with exposed risk time from first dispensed finasteride prescription. The number of men with MBC in the unexposed cohort was calculated as the difference between number of MBC in the general population and the exposed cohort.

Having ever redeemed finasteride was the primary exposure variable. Supplementary analyses of finasteride exposure were performed evaluating duration of exposure, cumulative dose, and time since first exposure. The cut‐off values for duration of finasteride exposure, dose, and time since first finasteride exposure were decided prior to analysis in consultation with specialists.

### Statistical analyses

The primary analysis investigated the association between having ever redeemed finasteride and the incidence rate of MBC among all men in the four countries and stratified by country. The interactions between age group at follow‐up and dispensed finasteride and between country and dispensed finasteride were evaluated. Further analyses on data from Denmark and Finland (with long follow‐up time) were performed evaluating the effect of duration of exposure, cumulative dose, and time since first exposure. The analyses were repeated after removing individuals with their first dispensed finasteride prescription in the first year of follow‐up (i.e., 1995 for Denmark and 1997 for Finland) to account for potential truncation bias (i.e., individuals with dispensed prescription in the first year might be prevalent users and the cumulative exposure cannot be correctly calculated).

All analyses were performed using a piecewise exponential model for survival data. The analysis was performed using a generalized linear model with a Poisson distribution of number of MBC cases and logarithmic transformation of risk time as offset value. Adjustment was made for age, calendar year, and country as fixed effects. Constant incidence rates within intervals of time were obtained by splitting the risk time for each country, 5‐year age group, and calendar year using the SAS Lexis macro 24. The incidence rate ratios (IRRs) of MBC for the exposed compared to the nonexposed and 95% CI were calculated. All analyses were performed using the PROC GENMOD procedure of SAS version 9.3 (SAS Institute Inc., Cary, NC).

### Sample size calculation

The minimum detectable IRR for MBC comparing finasteride exposed to nonexposed was calculated at 2.6 and 1.9, respectively, assuming a total population of approximately 12 mill men, 10.8 per 1000 men exposed to finasteride, an incidence rate (IR) of MBC among nonexposed at 0.4 and 1 per 100,000 PY, respectively, 15 years follow‐up, a 5% significance level, and a power of 80% 25.

## Results

During follow‐up, a total of 902 MBC cases occurred in 112 million PY in the male population in Denmark, Finland, Norway, and Sweden (Table 1). This corresponded to a crude IR of 0.80 MBC cases per 100,000 PY (Table 1). The IR per 100,000 varied between 0.92 in Denmark and 0.66 in Finland. The maximal follow‐up time in the four countries was 15, 14, 6, and 5 years in Denmark, Finland, Norway, and Sweden, respectively. Median time from initiation of finasteride use to MBC diagnosis was 40 months based on 55 MBC cases in Denmark and Finland with long follow‐up time.

**Table 1:** Number of male breast cancer cases (*N*_MBC_), number of person years (*N*_PY_), and crude and age‐standardized (Nordic Standard Population, 2000) incidence rate of MBC (IR) per 100,000 in 1995–2010 overall and stratified by country, calendar period, and age group

Variable	Level	*N*_MBC_	*N*_PY_	IR per 100,000	
				Crude	Age standardized
Overall		902	112,425,969	0.80	0.91
Country	Denmark	365	39,844,762	0.92	1.08
	Finland	236	35,834,189	0.66	0.74
	Norway	101	14,011,628	0.72	0.82
	Sweden	200	22,735,390	0.88	0.89
Year	1995–1998	116	15,424,184	0.75	0.98
	1999–2002	165	20,691,378	0.80	0.96
	2003–2006	303	36,821,669	0.82	0.90
	2007–2010	318	39,488,738	0.81	0.85
Age group	0–34	9	50,567,403	0.02	–
	35–39	14	8,288,627	0.17	–
	40–44	22	8,402,741	0.26	–
	45–49	33	8,120,563	0.41	–
	50–54	56	8,034,841	0.70	–
	55–59	85	7,512,897	1.13	–
	60–64	117	6,496,764	1.80	–
	65–69	117	4,915,216	2.38	–
	70–74	148	3,882,216	3.81	–
	75–79	116	2,964,162	3.91	–
	80–84	99	1,943,265	5.09	–
	≥85	86	1,296,790	6.63	–

In the last year of follow‐up, the PY among finasteride users accounted for 1.1% in Denmark, 1.4% in Sweden, 3.0% in Finland, and 0.7% in Norway out of PY in the total male populations (Fig. S1). During the study period, finasteride users of 5 mg packages constituted 89% of the PY among finasteride users in Denmark and 90% in Sweden in 2009.

A total of 63 men developed MBC after dispensed finasteride: 29 in Denmark, 26 in Finland, 1 in Norway, and 7 in Sweden (Table 2). There was a significantly increased incidence of MBC among finasteride users compared to nonusers with an IRR of 1.44 (95% CI = 1.11–1.88, *P* = 0.009). The IRR for dispensed finasteride increased to 1.60 (1.20–2.13) for the analysis of Denmark and Finland with long follow‐up time. The country‐specific IRR was highest in Denmark (IRR = 1.81, 95% CI = 1.23–2.67, *P* = 0.006). There was no significant interaction between country and dispensed finasteride (*P* = 0.18 for interaction term). The association between dispensed finasteride and MBC was modified by age at follow‐up with IRR at 5.58 (95% CI = 3.14–9.92) for age<70 years at follow‐up decreasing to 1.67 and 1.15 for age 70–79 years and ≥80 years at follow‐up, respectively (Table 2). When restricting the exposed group to finasteride users with 5 mg finasteride, a significant association was found (IRR = 1.60, 95% CI = 1.20–2.14).

**Table 2:** Association between dispensed finasteride and male breast cancer (MBC) in Denmark, Finland, Norway, and Sweden given by MBC cases, person years, *P*‐value, incidence rate ratio (IRR), and 95% confidence interval (95% CI) adjusted for differences in age, calendar year, and country

	Finasteride exposure	Cancer cases	Person years	Overall *P*‐value	IRR	95% CI
Primary analysis	User	63	1,365,088	0.009	1.44	1.11–1.88
	Nonuser	839	111,060,881		1	(ref)
Age group at follow‐up						
0–69 years	User	12	519,340	<0.001	5.58	3.14–9.92
	Nonuser	441	101,820,197		1	(ref)
70–79 years	User	30	511,253		1.67	1.14–2.44
	Nonuser	234	6,335,125		1	(ref)
≥80 years	User	21	334,494		1.15	0.73–1.81
	Nonuser	164	2,905,560		1	(ref)
Country specific						
Denmark	User	29	379,540	0.006	1.81	1.23–2.67
	Nonuser	336	39,465,222		1	(ref)
Finland	User	26	712,941	0.10	1.45	0.95–2.23
	Nonuser	210	35,121,248		1	(ref)
Norway	User	1	59,605	0.51	0.55	0.08–3.94
	Nonuser	100	13,952,023		1	(ref)
Sweden	User	7	213,002	0.92	0.96	0.45–2.06
	Nonuser	193	22,522,388		1	(ref)

Further analyses of Denmark and Finland showed that the risk of MBC was highest among men who redeemed finasteride up to 3 years (IRR = 1.89, 95% CI = 1.37–2.60) and among men whose accumulated consumption corresponded to up to three finasteride packs with 98 pills of 5 mg (IRR = 1.92, 95% CI = 1.33–2.79) (Table 3). The relative risk of MBC in finasteride users was highest during the follow‐up period of 1–3 years after first dispensed finasteride prescription (IRR = 2.28, 95% CI = 1.42–3.67), but not markedly lower during the first and fourth to fifth years of follow‐up (Table 3). The effect of duration of dispensed finasteride, accumulated dispensed finasteride prescriptions, and years after first dispensed finasteride prescription did not change when individuals with their first dispensed finasteride prescription in the first year of the prescription registers were excluded (Table 3).

**Table 3:** Association between dispensed finasteride, duration of dispensed finasteride, dose, and time since first dispensed finasteride prescription and male breast cancer (MBC) in Denmark and Finland given by MBC cases, risk time, *P*‐value, incidence rate ratio (IRR), and 95% confidence interval (95% CI). Results are adjusted for differences in age, calendar year, and country

Exposure categories	Including finasteride users with dispensed finasteride in first year of follow‐up					Excluding finasteride users with dispensed finasteride in first year of follow‐up				
	Cancer cases	Person years	Overall *P*‐value	IRR	95% CI	Cancer cases	Person years	Overall *P*‐value	IRR	95% CI
Dispensed finasteride										
User	55	1,092,481	0.002	1.60	1.20–2.13	33	793,483	0.10	1.37	0.95–1.96
Nonuser	546	74,586,470		1	(ref)	535	69,800,789		1	(ref)
Duration of dispensed finasteride (years)										
Nonuser	546	74,586,470	0.001	1	(ref)	535	69,800,789	0.09	1	(ref)
≤3	42	732,084		1.89	1.37–2.60	27	567,664		1.60	1.08–2.37
3.1–5	8	145,399		1.71	0.84–3.45	4	103,046		1.25	0.46–3.35
>5	5	214,998		0.66	0.27–1.60	2	122,773		0.48	0.12–1.95
Dose (categorized as number of finasteride packs with 98 pills of 5 mg)										
Nonuser	546	74,586,470	0.007	1	(ref)	535	69,800,789	0.14	1	(ref)
≤3	30	528,958		1.92	1.33–2.79	21	415,570		1.76	1.13–2.74
3.1–6	9	161,739		1.80	0.92–3.50	3	120,040		0.82	0.26–2.56
>6	16	401,784		1.15	0.69–1.91	9	257,873		1.04	0.53–2.02
Time since first finasteride purchase (years)										
Nonuser	546	74,586,470	0.01	1	(ref)	535	69,800,789	0.15	1	(ref)
<1	8	159,670		1.85	0.92–3.73	5	128,835		1.41	0.58–2.42
1–2.9	18	271,837		2.28	1.42–3.67	13	215,122		2.09	1.20–3.65
3–4.9	11	219,610		1.62	0.89–2.95	7	169,236		1.37	0.65–2.90
5+	18	441,363		1.16	0.72–1.87	8	280,290		0.85	0.42–1.73

## Discussion

In this population‐based register study, we found a statistically significant 44% increased risk of MBC among men having finasteride prescribed compared to nonusers. The IRRs were highest for males in Denmark and Finland, that is, countries with longest follow‐up period. The IRRs were highest in the categories of medium duration or lowest dose of finasteride which is against a causality of the association. An excess risk was identified in the first year after the first known dispensed finasteride and it decreased after 5 years. Such a short time lag and a decreasing risk over time are not typical in cancer causation. If the relative risk was first nonelevated and then increased after some years, it could be a sign of causal relation.

The main strength of this study is that it includes the largest number of persons exposed to finasteride reported yet. It has relatively long maximum follow‐up time especially for the Danish and Finnish cohorts, and contains information for the total population and individual‐level register data collected independently of the research question. The quality of the prescription and cancer registers is high due to the completeness and validity of registered data 26, 27, 28, 29.

There are some study limitations to be considered. First, exposure data were based on dispensed finasteride and not finasteride consumption. Exposure may therefore be overestimated, and the found association between dispensed finasteride and MBC may as a consequence be underestimated. Second, due to the rarity of MBC, the statistical power is limited. Third, surveillance bias may have influenced the results because physicians may follow men with dispensed finasteride more closely or because of breast complaint and development of gynecomastia may lead to closer surveillance of users than nonusers. The finding of higher risk of breast cancer among short‐term finasteride users could be a reflection of surveillance bias. Fourth, information about dispensed finasteride before establishment of the prescription registers was not available. Therefore, the analyses accounting for “years after first dispensed finasteride prescription,” “cumulative dose,” and “duration of dispensed finasteride” underestimate the association. However, excluding finasteride users dispensed in the first year of data collection, as we do in Table 3, in principle corrects for this error. Fifth, the number of persons followed up for longer time periods is still quite small and hence the power of the study to reveal possible role of finasteride as a cancer initiator is limited. If the finasteride would act as a moderator in the cancer process, then the lag from exposure to outcome is essentially shorter.

Most important limitation is the lack of data on potential confounding factors. Factors influencing the estrogen/testosterone balance, including testicular abnormality, benign breast disease, gynecomastia, estrogen therapy, occupational exposures, and physical inactivity, obesity, family history of breast cancer, and comorbidities such as liver disease and alcohol intake, can be associated with both dispensed finasteride and MBC thereby confounding the observed association 18, 23.

The crude incidence rate of MBC estimated in the present study at 0.8 per 100,000 PY is within the range 0.4–1.0 per 100,000 PY reported in earlier epidemiological studies 12, 13, 14. The median time from initiation of finasteride use to MBC diagnosis was 40 months in the present study. This is in accordance with previous studies, for example, MHRA reported a median time at male breast cancer developing 36 months after starting treatment with 5 mg finasteride for enlarged prostate 6, 7.

Conflicting results are seen for the association between finasteride use and MBC from the few published studies in this area. A number of studies have indicated a possible link between finasteride use and risk of MBC 3, 5, 6, 7, 8. Bird et al. (2013) reported no significant association, but the follow‐up time was only 1–3 years after exposure 10. This is considered a short follow‐up period. Duijnhoven et al. estimated a nonsignificant OR at 1.08 for the association between ever use of finasteride or dutasteride, but an OR at 1.29 for cumulative use for 3 or more years which is similar to the effect of cumulative use for 3–5 years in the present study with an IRR at 1.25 11.

Only few observational epidemiological studies have been performed and the estimated association between finasteride use and MBC varies between these studies. In general, the Nordic prescription databases constitute an outstanding resource for pharmacoepidemiological studies in large populations 30, and pharmacoepidemiological studies in the Nordic countries are considered to have high validity 31.

We conclude that this study showed a significant increased risk of MBC among men having finasteride prescribed compared to nonusers. The IRRs were highest for males in Denmark and Finland, that is, countries with longest follow‐up period. Due to limited data for adjustment of potential confounding, more research is warranted to confirm the results. AG and ME declare: They have received finance from Merck Sharp & Dohme Corp. for the submitted work.

## Conflicts of Interest

MM, LCT, KBr, PI, EP, KBo, KS, and AKE declare: no conflict of interest. AG and ME declare: They have received finance from Merck Sharp & Dohme Corp. for the submitted work. They declare no other relationships or activities that could appear to have influenced the submitted work. This article is based on data from a study conducted by Applied Economics and Health Research (ApEHR) as an independent research organization based on a regulatory request from the European Medicines Agency (EMA) and funded by Merck Sharp & Dohme Corp (MSD). MSD has had the opportunity to comment on the manuscript, but the authors retained the right to accept or reject comments and suggestions.

## Supporting information


**Figure S1.** Percentage of PY among finasteride users out of PY in the total male population in each of the four Nordic countries.
