# Supplementary material for: Finasteride treatment and male breast cancer: a register‐based cohort study in four Nordic countries
Source: Cancer Med. 2017 Dec 13;7(1):254–60. doi: 10.1002/cam4.1273 (PMC5773955; doi:10.1002/cam4.1273)
Supplement: Supplementary file 1 — Figure S1. Percentage of PY among finasteride users out of PY in the total male population in each of the four Nordic countries. [file CAM4-7-254-s001.docx]

**Online supplementary Figure 1.** Percentage of PY among finasteride users out of PY in the total male population in each of the four Nordic Countries.
